# Microorganism community composition analysis coupling with ^15^N tracer experiments reveals the nitrification rate and N_2_O emissions in low pH soils in Southern China

**DOI:** 10.1515/biol-2022-0010

**Published:** 2022-02-15

**Authors:** Feifei He, Haohao Yu, Dandan Liu, Zheng Li

**Affiliations:** School of Agriculture, Yunnan University, Kunming 650500, China

**Keywords:** acid soils, nitrification, ammonia-oxidizing organisms, ^15^N tracing

## Abstract

Nitrification in agricultural soil is an important process for food production. In acidic soil, nitrification is however also considered to be a major source of N_2_O production. The nitrification rate largely depends on the community composition of ammonia-oxidizing organisms. To obtain a view of the nitrification rates and N_2_O emission situations in low pH soils in Southern China and understand their relations with the microbial community composition, here we conducted ^15^N tracer experiments and microorganism community composition analysis using four acidic agricultural soil samples collected in Southern China. A single dominant community (relative abundance >68%) of the ammonia-oxidizing bacteria and ammonia-oxidizing archaea was observed in the soils with pH = 4.81–6.02. A low amount of {\text{NO}}_{3}^{\mbox{--}}] was produced from the nitrification in the strongly acidic soil (pH = 4.03), and the calculated nitrification rate in this soil was significantly lower than those of other soils with pH = 4.81–6.02. High N_2_O emissions but low ^15^N–N_2_O emissions were observed in the soil with pH = 4.03. Our results suggest that, under aerobic conditions, soil pH is an important factor affecting nitrification through modifying the microorganism composition.

## 1 Introduction

It is widely known that nitrogen is of utmost importance to plants. Therefore, routinely applied nitrogen-based fertilizers are necessary for maintaining agricultural production [1]. Nitrogen uptake in plants involves the biological oxidation of ammonium to nitrate via nitrite, in a process termed nitrification. In soil, there are two major categories of microorganisms responsible for this process, namely, autotrophic ammonia-oxidizing bacteria (AOB) [2,3,4] and ammonia-oxidizing archaea (AOA) [5,6]. With nitrite being the intermediate product, AOB carries out most ammonia oxidation in soil, which is the primary step in the oxidation process converting ammonia to nitrate and is considered the rate-limiting step of nitrification in most soil systems [7]. With regard to AOA, while they have also been reported to possess the ammonia monooxygenase α-subunit (*amoA*) gene, their ammonia oxidation pathway is less clear [8,9]. Furthermore, AOA’s genomes typically harbor a large number of *amoA* genes than AOB in many ecosystems [10,11]. Interestingly, Lu et al. [12] and Zhang et al. [13] reported that AOA might play a more important role in nitrification in acidic soils than AOB.

Nitrification is highly sensitive to soil pH. The suitable pH range for nitrification to take place in the soil is 5.5–10.0, with the optimal pH being around 8.5. In the 10 soils studied by Sahrawat [14], when the soil pH was less than 5.0, no nitrification was detected at all. In some rare cases, nitrification may also occur in soils with extremely low pH (e.g., 3.8), as reported by Tisdale and Nelson [15]. Nevertheless, strongly acidic soils generally have limited nitrification abilities. Although applying nitrogen-based fertilizers and/or manure can accelerate nitrification in acidic soil [16], such promoting effect is only moderate in highly acidic soil [17,18,19].

Nitrous oxide (N_2_O) is a greenhouse gas that contributes to the depletion of the stratospheric ozone layer. Agricultural and natural soils collectively give rise to approximately 50–70% of total global emissions [20]. Nitrification is one of the major processes that emit N_2_O in soil, especially under aerobic conditions. In addition, high N_2_O emissions stemming from denitrification were also observed in acidic soils under aerobic conditions [21].

The central hypothesis of this work was that the high nitrification rate in acidic soils is largely due to the specific dominant ammonia-oxidizing microbial communities. To verify this, we carried out microorganism community composition analysis coupling with ^15^N tracer experiments to reveal the effects of soil pH on the nitrification rate and N_2_O emissions and explored the underlying mechanism.

## 2 Materials and methods

### 2.1 Experimental soils

Soil samples were collected from four agricultural fields in Yunnan Province, Southern China (Table 1). Ten samples (0–0.2 m depth) were collected and pooled for each soil type. For soil property measurement, we followed the standard methods described in ref. [22]. Briefly, the soil samples were first air-dried and sieved through a 4 mm mesh. Subsequently, the soil was digested with potassium dichromate and concentrated sulfuric acid, and residual dichromate was titrated with FeSO_4_ (0.2 M) to determine the total soil organic carbon. The total soil N was estimated following the micro-Kjeldahl digestion–distillation procedure. Finally, the pH value was measured using a pH meter, following the procedure described in ref. [23].

**Table 1:** Information of sampling sites and soil properties

Soil	I	II	III	IV
Sampling site	Wenshan	Yuxi	Kunming	Wenshan
Coordinates	N 24°16′609′′	N 24°17′511′′	N 24^o^49′778′′	N 24°03′271′′
	E 104°51′788′′	E 102°22′505′′	E 102^o^50′279′′	E 105°04′910′′
Land use	Tea garden	Corn field	Vegetable field	Vegetable field
pH (water)	4.03	4.81	5.41	6.02
Total organic carbon (g C kg^−1^)	27.2	23.7	12.9	7.46
Total nitrogen (g N kg^−1^)	2.60	2.00	1.22	0.85
<2 µm clay particles (%)	75.0	71.0	74.3	23.6

### 2.2 DNA extraction and terminal restriction fragment length polymorphism (T-RFLP) analysis of the *amoA* genes

The same samples used for soil property measurement were used for DNA extraction. Immediately after the soil samples were collected and pooled, an appropriate amount of soil was quickly wrapped in aluminum foil, frozen in liquid nitrogen, and stored at −80°C. Genomic DNA was extracted from 0.5 g of frozen soil using the HiPure Soil DNA Mini Kit (Magen Bio Inc., Guangzhou, China) following the manufacturer’s instructions. The concentration and purity of the extracted DNA were assessed using the Biophotometer plus system (Eppendorf, Hamburg, Germany). For T-RFLP analysis, PCR amplifications were performed using the primer pairs Arch-amoAF/Arch-amoAR (for AOA) [24] and amoA1F/amoA2R (for AOB) [25]. Each forward primer was fluorescently labeled using 5-carboxyfluorescein. The thermocycling PCR conditions were 94°C for 2 min followed by 30 cycles of 94°C for 20 s, 57°C for 45 s, and 72°C for 45 s. The PCR products were electrophoresed on 1.0% (m/v) agarose gels and detected using an image analyzer (UV/white transilluminator). Subsequently, the PCR products were gel-purified using the Agarose Gel Extraction Kit (Tiangen Inc., Beijing, China) and digested using the restriction enzyme *Taq*I (Takara Bio Inc., Shiga, Japan). The mixture (17 µL of the purified PCR products, 2 µL of buffer, and 1 µL of 10 U/µL *Taq*I) was incubated at 37°C for 4 h. The terminal restriction fragments (T-RFs) of AOA and AOB were fluorescently labeled by Sangon Inc. (Shanghai, China). The relative abundance of each T-RF was determined by calculating the ratio of the area of each fluorescence peak to the total area.

### 2.3 ^15^N-tracer experiments

The nitrification rate was estimated according to the final pool size of {\text{NO}}_{3}^{\mbox{--}}] that was derived from labeled {\text{NH}}_{4}^{+}] . This estimated nitrification rate may be slightly lower than the actual rate as the removal of {\text{NO}}_{3}^{\mbox{--}}\hspace{.25em}] as denitrification was not taken into account [26]. Briefly, the ammonium pool was labeled using (^15^NH_4_)_2_SO_4_ (10.13 atom% excess). Air-dried soils were adjusted to 45% of the soil’s water-holding capacity and preincubated aerobically at 25°C in the dark for 7 days before use. For each soil sample, 18 Erlenmeyer flasks (250 mL) each containing 80 g of the soil (oven-dried) were prepared. About 1 mL of (^15^NH_4_)_2_SO_4_ solution was added to each flask at a concentration of 50 mg NH_4_–N kg^−1^. The soil-(^15^NH_4_)_2_SO_4_ mixture was then adjusted to 60% of its water holding capacity and incubated for 7 days at 25°C. The soils (three replications) were extracted at 2 h and 1, 2, 3, 5, and 7 days after the addition of (^15^NH_4_)_2_SO_4_. The concentrations and isotopic compositions of both {\text{NH}}_{4}^{+}-N] and {\text{NO}}_{3}^{\mbox{--}}\text{–}N] were measured by using a continuous flow analyzer (AA3, SEAL, Germany) and a PDZ Europa 20-22 isotope ratio mass spectrometer (IRMS, SerCon, Crewe, UK), respectively. Gas samples were collected at 2 h and 1, 2, 3, 5, and 7 days after adding (^15^NH_4_)_2_SO_4_ to the soil. Gas samples (40 mL) were collected from each Erlenmeyer flask and then injected into two pre-evacuated vials (18.5 mL), one for determining the concentration with an Agilent 7890 gas chromatogram and the other for measuring the isotopic composition of N_2_O.

### 2.4 Calculation and statistical analyses

Nitrification rates were calculated as described by Mørkved et al. [26]:

c\left=[{{(}^{\ast }{\text{NO}}_{3}^{\mbox{--}})}_{t}\hspace{0.25em}\mbox{--}\hspace{0.25em}{{(}^{\ast }{\text{NO}}_{3}^{\mbox{--}})}_{0}]/{[}{(}^{\ast }{\text{NH}}_{4}^{+})\hspace{.25em}\left\mbox{--}\hspace{0.25em}\text{}{{(}^{\ast }{\text{NO}}_{3}^{\mbox{--}})}_{0}],]

where *c* is the relative share of {\text{NO}}_{3}^{\mbox{--}}-N] (originating from {\text{NH}}_{4}^{+}-N] ) at the end of the experiment; {({\text{⁎NO}}_{3}^{\mbox{--}})}_{t}] is the atom% ^15^N in {\text{NO}}_{3}^{\mbox{--}}] at the end of the experiment; {(\ast {\text{NO}}_{3}^{\mbox{--}})}_{0}] is the atom% ^15^N in {\text{NO}}_{3}^{\mbox{--}}] at the start of the experiment; and (\ast {\text{NH}}_{4}^{+})] is the average atom% ^15^N in {\text{NH}}_{4}^{+}] during incubation. To estimate the nitrification rate, *c* was multiplied by the {\text{NO}}_{3}^{\mbox{--}}] concentration at the end of incubation and divided by the incubation time.

The modeled nitrification rates were calculated using the following equation (on the basis of the changes in the ^{15}{\text{NO}}_{3}^{\mbox{--}}] content along with incubation):

{N}_{{\text{NO}}_{3}}={N}_{0}+{k}_{0}t,]

where *N*_NO3_ is the ^{15}{\text{NO}}_{3}^{\mbox{--}}] content at incubation time *t*, *N*_0_ is the ^{15}{\text{NO}}_{3}^{\mbox{--}}] content at the start of the experiment, and *k*_0_ is the rate constant of the zero-order reaction.

Multiple comparisons were made using one-way ANOVA with Duncan’s post-hoc test. All analyses were conducted using the SPSS 25.0 package (SPSS Inc., Chicago, USA), and *p* < 0.05 was considered to be statistically significant.

## 3 Results

### 3.1 Soil properties

Soil I (pH = 4.03) contained a total organic carbon content of 27.2 g C kg^−1^ and a total nitrogen content of 2.60 g N kg^−1^. The soil was sampled in late spring from the plough layer of a field where tea plants were grown for ∼20 years. Soil II (pH = 4.81), obtained from a corn–corn rotation field, contained a total organic carbon content of 23.7 g C kg^−1^ and a total nitrogen content of 2.00 g N kg^−1^. Soil III (pH = 5.41) and soil IV (pH = 6.02) were obtained from a vegetable planting field, and their total organic carbon and total nitrogen contents were estimated as 12.9 and 7.46 g C kg^−1^, and 1.22 and 0.85 g N kg^−1^, respectively (Table 1).

### 3.2 T-RFLP analysis of AOA and AOB

**Figure 1:**
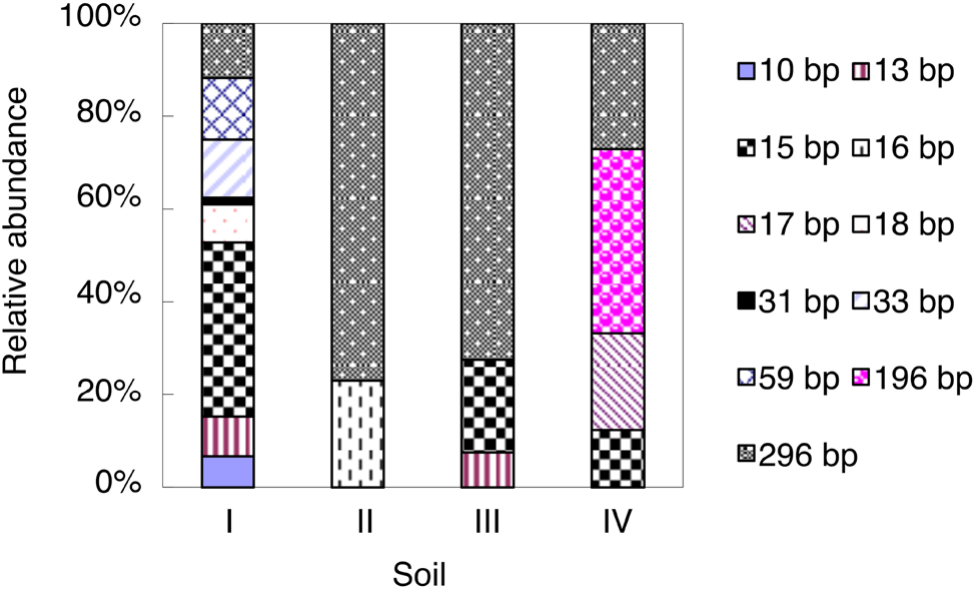
Relative abundance of AOB *amoA* T-RFs in the studied soils at the end of the incubation. For soil information, see Table 1.

As shown by the AOB T-RFLP profiles, the 296-bp T-RF was the most dominant AOB T-RF in soils II and III and accounted for 73–77% of the total AOB T-RFs, while it showed low relative abundance (12–27%) in soils I and IV (Figure 1). Meanwhile, the 15-bp T-RF in soil I and the 196-bp T-RF in soil IV were the dominant AOB T-RFs and accounted for 38 and 40% of the total AOB T-RFs, respectively (Figure 1). In soils II, III, and IV, only two (16- and 296-bp), three (13-, 15-, and 296-bp) and four (15-, 17-, 196-, and 296-bp) T-RFs were detected, respectively, whereas as many as eight T-RFs were detected in soil I (Figure 1).

**Figure 2:**
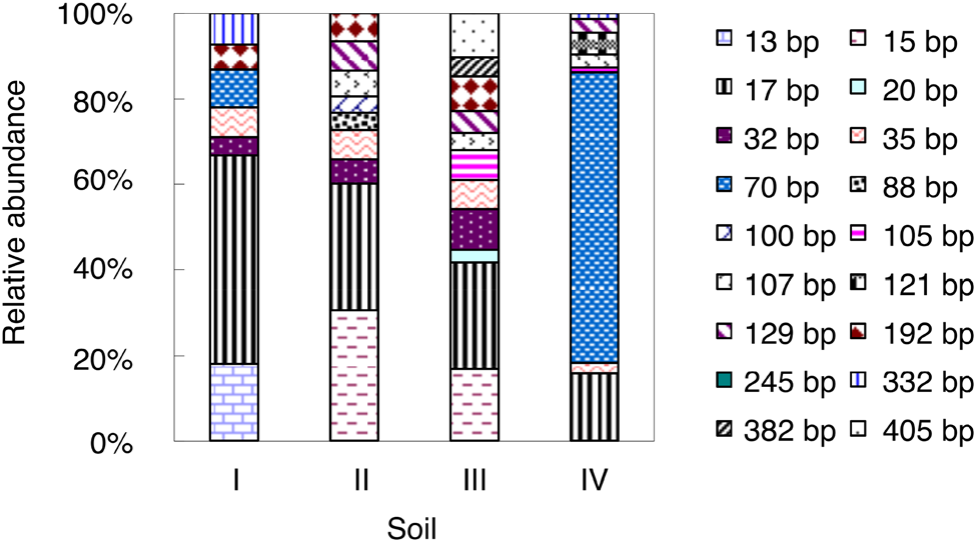
Relative abundance of AOA *amoA* T-RFs in the studied soils at end of the incubation. For soil information, see Table 1.

As shown in Figure 2, 18 AOA T-RFs were detected in the studied soils. Such a large number suggested that the AOA communities had relatively higher diversity than the AOB communities. The 70-bp T-RF was the most dominant AOA T-RF and accounted for 68% of the total AOA T-RFs. Notably, this T-RF was only detected in soils I and IV.

### 3.3 Soil inorganic N

**Figure 3:**
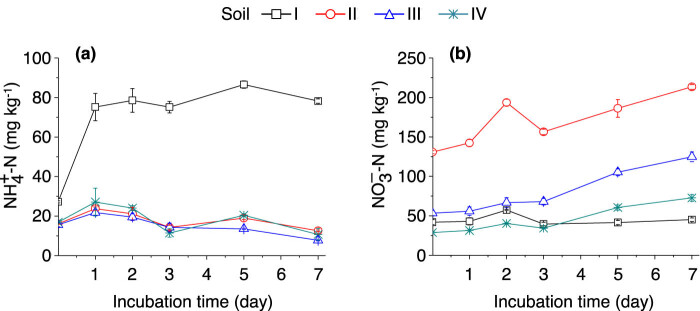
Dynamics of soil {\text{NH}}_{4}^{+}-N] (a) and {\text{NO}}_{3}^{\mbox{--}}\text{–}N] (b) content after adding (^15^NH_4_)_2_SO_4_. The error bars represent SEM. *n* = 3 replicates. For soil information, see Table 1.

**Figure 4:**
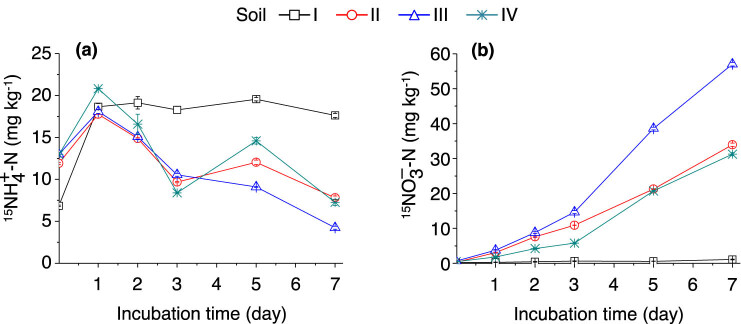
Dynamics of soil {}^{15}N{H}_{4}^{+}-N] (a) and {}^{15}N{\text{O}}_{3}^{\mbox{--}}\text{–}N] (b) content after adding of (^15^NH_4_)_2_SO_4_. The error bars represent SEM. *n* = 3 replicates. For soil information, see Table 1.

The ammonium concentration of soil I increased rapidly after being incubated with (^15^NH_4_)_2_SO_4_, and following 1 day of incubation, the ammonium concentration in soil I was constantly higher than those in soils II–IV (Figure 3a). {\text{NH}}_{4}^{+}-N] concentration showed no change in soil I during the following days but significantly decreased in soils II–IV, especially at the end of the incubation. By contrast, {}^{15}N{H}_{4}^{+}-N] concentration rapidly increased in the four soils at the early time points after being incubated with (^15^NH_4_)_2_SO_4_ and then significantly decreased in soils II–IV. Compared with other soil samples, soil I had the highest average {}^{15}N{H}_{4}^{+}-N] concentration and it did not significantly fluctuate during the incubation period. After 7 days of incubation, approximately 35% of the added (^15^NH_4_)_2_SO_4_ was detected in the NH_4_^+^ pool in soil I, whereas less than 16% was detected in soils II–IV (Figure 4a). With regard to the {\text{NO}}_{3}^{\mbox{--}}\text{–}N] concentration, the highest average value was found in soil II during the incubation period (Figure 3b). The {\text{NO}}_{3}^{\mbox{--}}\text{–}N] concentration increased in all soil samples after being incubated with (^15^NH_4_)_2_SO_4_, with the increase being more significant in soil II but only moderate in soil I (Figure 3b). The fact that {}^{15}N{O}_{3}^{\mbox{--}}] concentration significantly increased along with (^15^NH_4_)_2_SO_4_ incubation time in soils II–IV suggested that the {\text{NO}}_{3}^{\mbox{--}}] was indeed produced from nitrification in the studied soils (Figure 4b). Interestingly, the {\text{NO}}_{3}^{\mbox{--}}] concentrations in soil I were significantly lower than those in soils II–IV during the incubation period, indicating that the oxidation of {\text{NH}}_{4}^{+}] to {\text{NO}}_{3}^{\mbox{--}}] was inhibited in soil I (Figure 4b).

**Figure 5:**
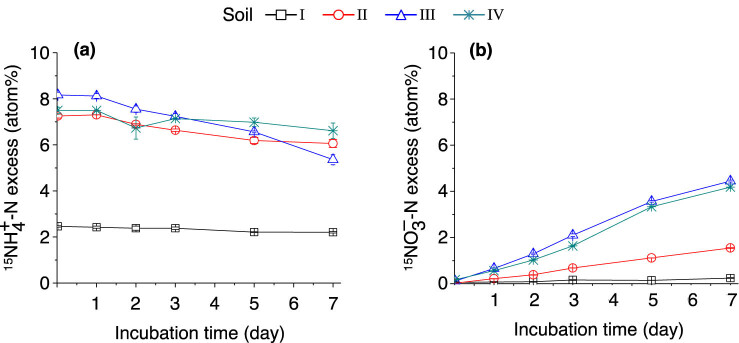
Dynamics of soil {}^{15}N{H}_{4}^{+}-N] atom% excess (a) and {}^{15}N{\text{O}}_{3}^{\text{–}}\text{–}N] atom% excess (b) after adding (^15^NH_4_)_2_SO_4_. The error bars represent SEM. *n* = 3 replicates. For soil information, see Table 1.

In the (^15^NH_4_)_2_SO_4_-labeled samples, the % ^15^N excess of the {\text{NH}}_{4}^{+}] pool gradually decreased over time in soils II–IV because of dilution by the mineralization of native soil organic N (Figure 5a). By contrast, due to the introduction of {\text{NO}}_{3}^{\mbox{--}}] derived from labeled {\text{NH}}_{4}^{+}] via nitrification, the % ^15^N excess of the {\text{NO}}_{3}^{\mbox{--}}] pool gradually increased in soils II–IV during the following days after being incubated with (^15^NH_4_)_2_SO_4_ for 2 h. In soil I, the % ^15^N excess of the {\text{NO}}_{3}^{\mbox{--}}] pool remained constant in soil I during the entire incubation period; approximately 53–65% of ^15^N was detected in the {\text{NH}}_{4}^{+}] pool after 7 days of incubation in soils II–IV, whereas only 22% was detected in soil I (Figure 5).

### 3.4 Nitrification rates

The lowest nitrification rate (0.52 mg N kg^−1^ day^−1^) was observed in soil I, yet the nitrification rate did not increase along with the pH gradient in soils II–IV (Table 2). Among the tested soil samples, soil III (pH = 5.41) displayed the highest nitrification rate. These results suggested that nitrification was significantly suppressed in soil I. As expected, the modeled nitrification rates were lower than the calculated nitrification rates, and a significant correlation was observed (*y* = −0.214 + 0.747*x*, *r* ^2^ = 0.996, *p* < 0.01).

**Table 2:** Nitrification rates of the studied soils

Soil	Calculated nitrification* (mg N kg^−1^ day^−1^)	Modeled nitrification** (mg N kg^−1^ day^−1^)
I	0.52 ± 0.03^d^	0.11 ± 0.02^c^
II	7.01 ± 0.10^b^	4.81 ± 0.06^b^
III	11.6 ± 0.57^a^	8.44 ± 0.10^a^
IV	6.05 ± 0.34^c^	4.62 ± 0.06^b^

Different letters denote significant differences (*p* < 0.05) among the studied soils. *Calculated according to the equation described by Mørkved et al. [26]. **Calculated following the zero-order equation.

### 3.5 N_2_O emissions

**Figure 6:**
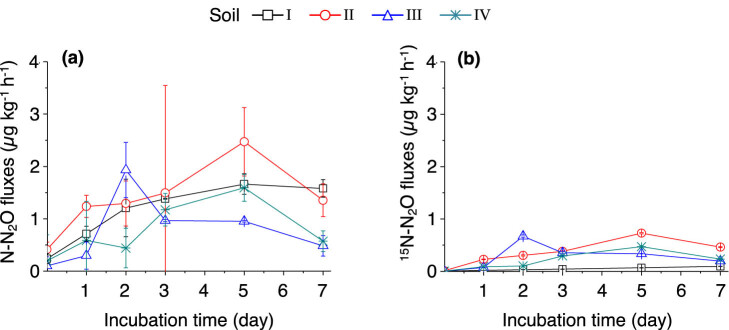
Dynamics of soil N_2_O emissions (a) and ^15^N–N_2_O emissions (b) after adding (^15^NH_4_)_2_SO_4_. The error bars represent SEM. *n* = 3 replicates. For soil information, see Table 1.

The fluxes of N_2_O in the studied soils are shown in Figure 6a. The N_2_O fluxes in soils I and II remained high during the incubation period and peaked on day 5. Between days 5 and 7, the N_2_O flux in soil I only slightly decreased while the flux in soil II dropped dramatically. The average N_2_O fluxes are shown in Table 3. Two highest average N_2_O fluxes were found in soil II (1.34 µg N kg^−1^ h^−1^) and soil I (1.11 µg N kg^−1^ h^−1^). The flux of ^15^N_2_O was higher in soil II than in soils I, III, and IV during the incubation period (Figure 6b). The ^15^N–N_2_O flux only slightly increased in soil I after the addition of (^15^NH_4_)_2_SO_4_, whereas the increases were significant in soils II–IV, suggesting that the oxidation of {\text{NH}}_{4}^{+}] to {\text{NO}}_{3}^{\mbox{--}}] was inhibited (Figure 6b).

**Table 3:** Average N_2_O flux and cumulative emissions from 2 h to 7 days after adding (^15^NH_4_)_2_SO_4_

Soil	N_2_O flux (µg N kg^−1^ h^−1^)	^15^N_2_O flux (ng N kg^−1^ h^−1^)	N_2_O emission (µg N kg^−1^)	^15^N_2_O emission (µg N kg^−1^)
I	1.11 ± 0.18^ab^	41.1 ± 2.48^d^	195 ± 30.3^ab^	6.19 ± 0.56^d^
II	1.34 ± 0.15^a^	351 ± 7.92^a^	255 ± 34.7^a^	66.0 ± 1.69^a^
III	0.72 ± 0.06^b^	271 ± 1.53^b^	138 ± 8.82^b^	50.9 ± 0.80^b^
IV	0.74 ± 0.12^b^	197 ± 5.29^c^	159 ± 39.5^ab^	41.1 ± 1.73^c^

Different letters denote significant differences (*p* < 0.05) among the studied soils.

During the 7 day (^15^NH_4_)_2_SO_4_ incubation period, the total N_2_O emissions in soils I and II were significantly higher (*p* < 0.05) compared with those of soils III and IV (Table 3). This result indicates that a low pH may negatively correlate with N_2_O emissions in soil. The ^15^N–N_2_O emission in soil I significantly decreased along with the incubation period, which is likely because the contribution of autotrophic nitrification to N_2_O production was suppressed in strongly acidic soil.

**Figure 7:**
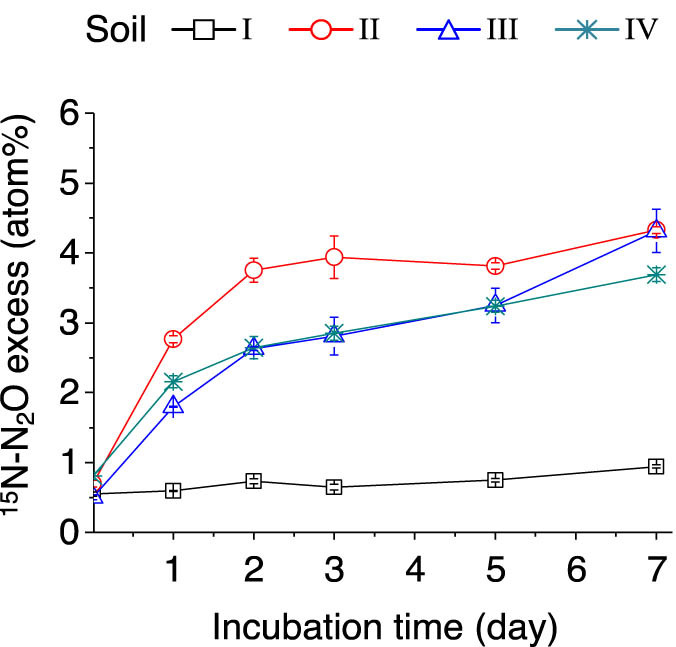
Dynamics of soil ^15^N–N_2_O atom% excess after adding (^15^NH_4_)_2_SO_4_. The error bars represent SEM. *n* = 3 replicates. For soil information, see Table 1.

In the (^15^NH_4_)_2_SO_4_-labeled samples, the % ^15^N excess of N_2_O gradually increased over time in soils II–IV because of the nitrification and/or denitrification of the labeled {\text{NH}}_{4}^{+}] , but it remained constant in soil I during the entire incubation period (Figure 7). These results further indicate that N_2_O production caused by autotrophic nitrification could be inhibited in strongly acidic soil.

## 4 Discussion

Soil pH is a key factor that controls the nitrification rate, and low pH conditions suppress nitrification [27,28,29]. In the present study, the nitrification rate in soil I (pH 4.03) was 91–95% lower than those in soils II–IV (pH 4.81–6.02). Given that the {}^{15}N{H}_{4}^{+}] content in soil I was high during the entire incubation period, it was likely that the pH condition rather {\text{NH}}_{4}^{+}] suppressed its nitrification activity. Our results are in agreement with the findings by Zhao et al. that high {\text{NH}}_{4}^{+}-N] concentrations do not inhibit nitrification rates in acidic soils [28]. The preferred form of ammonium for ammonium-oxidizing organisms is NH_3_ [29]. When the pH is low, a large amount of NH_3_ is ionized to form {\text{NH}}_{4}^{+}] , which is considered to be the major reason for the reduced activity of ammonia oxidation at low pH conditions [30,31]. We hypothesize that the nitrification rates are strongly dependent on the soil pH and that it is presumably through controlling the NH_3_ concentration, although the direct relation between pH and ammonia concentration was not investigated in this study.

In addition to substrate limitation, our finding that the most acidic soil (soil I, pH = 4.03) displayed the lowest nitrification rate might also be explained by the low abundance and/or activity of AOA [13,32,33]. qPCR quantification of AOB and AOA showed no significant difference between these two in terms of the total microbial amount in the studied soils (data not shown). However, our T-RFLP results revealed compositional variations of the AOB and AOA communities in the four studied soils. The AOB communities were relatively less diversified compared with the AOA communities in this study (Figures 1 and 2). The T-RFLP profile showed that only one AOB T-RF (296 bp) remained dominant in soils II and III at the end of the incubation period. No dominant AOB T-RF was detected in soils I and IV. By contrast, the AOA communities featured a dominant 70-bp T-RF, except for the community in soil IV. Our results suggest that dominant communities, rather than community abundance, may exert a major effect on nitrification. In our study, a single dominant T-RF of AOB and/or AOA may contribute to the higher nitrification rates observed in soils II–IV. It is worth noting that, in addition to soil pH, the field management practice is another factor influencing microbial composition. Notably, it has been reported that AOA and AOB could respond differently to management practices [23]. In the four soil samples we studied, only soil I was from a perennial system, which was less fertilized than other annual cropping systems (soils II–IV). Thus, nitrogen input differences may also contribute to the varied niche differentiation between AOA and AOB in the four soils.

Our N_2_O emission results suggest that soil N_2_O emissions are enhanced under low pH conditions. This is in line with the findings of Van den Heuvel et al. [34]. In their study, only 25% of the soil spots were of low pH (<5), but these soil spots gave rise to 77% of the total N_2_O emission. In the present study, the low pH value in soil I resulted in both higher N_2_O flux and emission. However, soil I’s flux and emission of ^15^N_2_O were lower than those of soils II–IV after adding (^15^NH_4_)_2_SO_4_. Hence, the N_2_O emission from strongly acidic soil may mainly be produced by denitrification, which is also an important process in a low pH environment. A previous ^15^N tracer experiment unraveled that, under aerobic conditions, denitrification in acidic soils could contribute to N_2_O production more markedly in comparison with autotrophic nitrification and heterotrophic nitrification [35]. Furthermore, an analysis based on 107 measurements in 26 publications also showed that in soils with a pH value lower than ∼4.4 and under aerobic conditions, denitrification is responsible for >50% of the soil N_2_O production [36]. From a chemical point of view, the N_2_O reductase produced during denitrification is sensitive to soil pH, and potential denitrifying enzyme activity is the highest in alkaline soil and the lowest in acidic soil [37]. Moreover, under low pH conditions, the reduction of N_2_O to N_2_ could be halted until {\text{NO}}_{3}^{\mbox{--}}] is depleted, resulting in N_2_O accumulation [34,38]. In addition to denitrification, heterotrophic nitrification may also be a significant source of N_2_O emission under low pH and aerobic conditions [39,40].

In summary, our results suggest that the nitrification rate and N_2_O emission are largely affected by soil pH by modifying the composition of AOB and/or AOA. Future work is needed to further characterize the AOB and AOA reported here and investigate if the high N_2_O emission observed in the acidic soil under aerobic conditions was mainly caused by denitrification.
